# A surrogate FRAX model for Pakistan

**DOI:** 10.1007/s11657-021-00894-w

**Published:** 2021-02-17

**Authors:** G. Naureen, H. Johansson, R. Iqbal, L. Jafri, A. H. Khan, M. Umer, E. Liu, L. Vandenput, M. Lorentzon, N. C. Harvey, E. V. McCloskey, J. A. Kanis

**Affiliations:** 1 Faculty of Medicine, Dentistry and Health Sciences, University of Melbourne, Melbourne, Australia; 2 Mary McKillop Institute for Health Research, Australian Catholic University, Melbourne, Australia; 3 Centre for Metabolic Bone Diseases, University of Sheffield, Sheffield, UK; 4 Departments of Community Health Sciences and Medicine, Aga Khan University, Karachi, Pakistan; 5 Department of Pathology & Laboratory Medicine, Aga Khan University, Karachi, Pakistan; 6 Department of Orthopaedics, Aga Khan University, Karachi, Pakistan; 7 Centre for Bone and Arthritis Research, Department of Internal Medicine and Clinical Nutrition, Institute of Medicine, Sahlgrenska Academy, University of Gothenburg, Gothenburg, Sweden; 8 Geriatric Medicine, Institute of Medicine, University of Gothenburg, Gothenburg, Sweden; 9 MRC Lifecourse Epidemiology Unit, University of Southampton, Southampton, UK; 10 NIHR Southampton Biomedical Research Centre, University of Southampton and University Hospital Southampton NHS Foundation Trust, Southampton, UK; 11 Mellanby Centre for Bone Research, Department of Oncology and Metabolism, University of Sheffield, Sheffield, UK

**Keywords:** FRAX, Fracture probability, Epidemiology, Hip fracture, Singaporean Indians, Surrogate, Pakistan

## Abstract

***Summary*:**

A surrogate FRAX® model for Pakistan has been constructed using age-specific hip fracture rates for Indians living in Singapore and age-specific mortality rates from Pakistan.

**Introduction:**

FRAX models are frequently requested for countries with little or no data on the incidence of hip fracture. In such circumstances, the International Society for Clinical Densitometry and International Osteoporosis Foundation have recommended the development of a surrogate FRAX model, based on country-specific mortality data but using fracture data from a country, usually within the region, where fracture rates are considered to be representative of the index country.

**Objective:**

This paper describes the development and characteristics of a surrogate FRAX model for Pakistan.

**Methods:**

The FRAX model used the ethnic-specific incidence of hip fracture in Indian men and women living in Singapore, combined with the death risk for Pakistan.

**Results:**

The surrogate model gave somewhat lower 10-year fracture probabilities for men and women at all ages compared to the model for Indians from Singapore, reflecting a higher mortality risk in Pakistan. There were very close correlations in fracture probabilities between the surrogate and authentic models (*r* ≥ 0.998) so that the use of the Pakistan model had little impact on the rank order of risk. It was estimated that 36,524 hip fractures arose in 2015 in individuals over the age of 50 years in Pakistan, with a predicted increase by 214% to 114,820 in 2050.

**Conclusion:**

The surrogate FRAX model for Pakistan provides an opportunity to determine fracture probability within the Pakistan population and help guide decisions about treatment.

## Introduction

In 2008, the then WHO Collaborating Centre for Metabolic Bone Diseases at the University of Sheffield, UK, launched the FRAX® tool for the calculation of 10-year fracture probabilities in women and men from readily obtained clinical risk factors (CRFs) and bone mineral density (BMD) measurements at the femoral neck (http://www.shef.ac.uk/FRAX). The algorithm (FRAX) was based on a series of meta-analyses using primary data from population-based cohorts that examined a list of candidate clinical risk factors for fracture [1, 2]. The output of FRAX comprises the probability of major osteoporotic fracture (hip, spine, distal forearm or proximal humerus) or hip fracture. This probability is in turn dependent upon the risk of fracture and the competing risk of death, both of which vary from country to country [3]. Ideally, data for age-specific incidences of fracture and death should be available for the construction of country-specific FRAX models, but information on fracture incidence is frequently poor or absent. On a positive note, the availability of FRAX has stimulated studies of fracture incidence that can be used for the generation of new FRAX models; specific examples include Armenia, Belarus, Brazil, Kazakhstan, Mexico, Russia, Turkey and Uzbekistan [4].

Recognizing that data on hip and other fractures are not always available, the International Society for Clinical Densitometry and International Osteoporosis Foundation recommend the development of a surrogate FRAX model to be used until country-specific data are collected and made available. Such surrogate models are based on age- and sex-specific mortality data from the index country, combined with age-specific, sex-specific rates of fracture derived from a country, usually nearby, where fracture rates are considered to be representative of the index country [5]. Of the 73 countries for which a FRAX model is available, six FRAX country-specific models currently use surrogate data on fracture risk (Georgia, India, Kyrgystan, Palestine, Sri Lanka and Syria). In the absence of good epidemiological data on fracture [6], the present report describes the development of a surrogate FRAX model for Pakistan.

## Methods

Pakistan is bordered by India to the east, China to the north, Afghanistan to the northwest, Iran to the west and a coastline along the Arabian Sea and Gulf of Oman in the south. Pakistan has an area of 881,913 km^2^ with a population estimated at 220,892,340 in 2020 [7, 8]. The population of Pakistan is young with a median age of 22.8 years, compared, for example, to a median age of 40.3 years in the UK [9].

### Development of surrogate model for Pakistan

Data on hip fracture risk were those derived for the population categorized as of Indian ethnicity in Singapore. The data have been used previously in the development of a surrogate FRAX model for India [4, 10]. Details of the FRAX model for Singapore are available elsewhere [11]. As described previously, in the absence of incidence data for other sites of major osteoporotic fracture (clinical spine, distal forearm and proximal humerus), the hip fracture rates were used to estimate these incidences on the assumption that the ratio of hip fracture incidence to these other FRAX outcomes is the same in the index country as that documented in Sweden, Iceland, Canada, Moldova and elsewhere [12–15]. National mortality rates for Pakistan used data from the World Health Organization for 2015–2019 [16].

### Comparative performance of the surrogate Pakistan FRAX model

For the purpose of comparing the authentic FRAX model for Singapore with the surrogate model for Pakistan, the probabilities of a major osteoporotic fracture (hip, clinical spine, forearm and humeral fractures) and of hip fracture alone were computed in men and women at ages 50, 60, 70 and 80 years for all possible combinations of clinical risk factors at BMD T-scores between 0 and −3.5 SD in 0.5 SD steps with a BMI set to 26 kg/m^2^ [17, 18]. This combination of six risk factors and eight values of BMD gave a total of 512 combinations at each age studied. Note that this was not a population simulation, but an array of all possible combinations. The correlation between the probabilities derived from the surrogate and authentic models was examined by piecewise linear regression with knots at the probabilities of 35% for the Singaporean Indian probabilities of a major osteoporotic fracture and hip fracture. Tabular data were used to compare probabilities between the two versions at the 50th (median) percentile of the distribution of the Singapore Indian model. Differences in the Pakistan model from the Singapore Indian model at these percentiles were expressed as 95% tolerance intervals (TI), analogous to a confidence interval but applied to individual cases.

The age- and sex-specific incidence was applied to the Pakistan population in 2015 to estimate the number of hip fractures nationwide in that year. Additionally, future projections were estimated up to 2050 assuming that the age- and sex-specific incidence remained stable. Population demography was taken from the United Nations using the medium variant for fertility [8].

## Results

Using the combinations of CRFs and BMD, the median probabilities for Pakistan were similar to those for Indians in Singapore for the age of 50 years, but with increasing age, the median values were lower in the Pakistan model, an effect that was more marked for men (Table 1). For example, in men at the age of 70 and 80 years, the median value of the surrogate version was lower by about 30% for the probability of hip fracture and major osteoporotic fracture, whereas at younger ages, the difference was less than 12% (Table 1). For women, the difference was less than 7% for ages below 70 years, but ranged from 11 to 23% lower values at the ages of 70 and 80 years.

**Table 1:** Probability (%) of a major osteoporotic fracture (MOF) or a hip fracture (with 95% tolerance intervals; TI) in men and women at the median of the probability distribution (Singapore version) by age. The *r* value provides the age-specific correlation coefficient between the Singaporean and Pakistani probabilities together with the 95% tolerance intervals (TI)

	Men			Women		
	Singapore	Pakistan		Singapore	Pakistan	
Age	Median		95% TI	Median		95% TI
MOF						
50	6.0	5.9	5.8–6.0	6.2	6.1	6.0–6.2
60	12.2	11.0	10.4–11.6	14.2	13.2	12.7–13.8
70	19.6	14.2	12.5–15.8	22.9	19.2	17.6–20.7
80	19.0	13.6	12.5–14.7	25.0	22.3	20.6–23.9
Hip fracture						
50	1.8	1.8	1.7–1.8	1.2	1.2	1.1–1.2
60	4.2	3.7	3.3–4.1	3.2	3.0	2.7–3.2
70	10.5	7.4	6.2–8.6	8.4	7.1	6.1–8.1
80	14.0	9.9	8.9–10.8	14.7	11.3	10.0–12.6

**Fig. 1:**
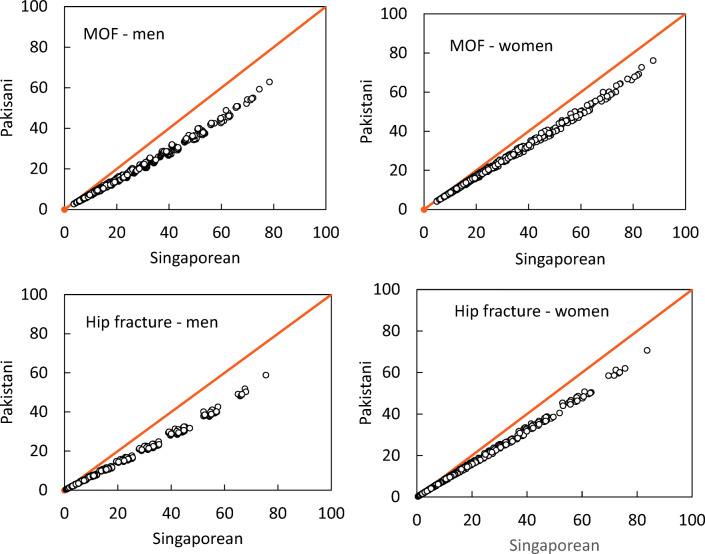
Comparison of 10-year probability of fracture using the surrogate Pakistan FRAX tool and the Singapore Indian FRAX tool for combinations of clinical risk factors and BMD at the age of 70 years. The left-hand panels show the comparison in men. The top panels relate to major osteoporotic fracture (MOF) and the lower panels to hip fracture probability. The diagonal line shows the line of identity

Despite differences in absolute values of probability, there was a close correlation between the FRAX model for Singapore and the surrogate Pakistan model. For all ages the correlation coefficients between the probabilities within risk factor combinations were high (*r* ≥ 0.998). The relationships between the probabilities of a major osteoporotic fracture and hip fracture derived from the two models of FRAX are shown for men and women age 70 years in Fig. 1.

### Fracture projections

Assuming that the fracture rates derived from Indians living in Singapore were representative for Pakistan, and based on the United Nations estimates of the Pakistan population for 2015, we estimated that the annual number of hip fractures in men and women age 50 years or older in Pakistan in 2015 totalled 36,524, comprising 12,902 hip fractures in men and 23,622 in women. The number of hip fractures is estimated to increase progressively by calendar year with an increase of 214% by 2050 (Table 2). The increase in hip fracture numbers is particularly high in women (231% in women and 184% in men) due to the longer life expectancy in women.

**Table 2:** Estimated total number of hip fractures (ICD-10 codes S72.0, S72.1, S72.2) in men and in women age 50 years and older in 2015 projected up to 2050 in Pakistan

	2015	2020	2030	2040	2050
Men	12,902	14,885	19,699	26,943	36,587
Women	23,622	27,364	37,948	54,840	78,233
Total	36,524	42,249	57,647	81,783	114,820
Increase (%)	-	16	58	123	214

## Discussion

This paper describes the development of a surrogate FRAX model for Pakistan, utilizing hip fracture rates from the ethnic Indian population of Singapore and mortality data from Pakistan. The surrogate model provided lower estimates of fracture probability for both major osteoporotic and hip fractures in men and women in Pakistan compared with the Singapore Indian model. The lower probabilities in Pakistan reflect differences in age-specific mortality between the two countries. Importantly, the differences had little impact on the stratification of risk, since there was little or no change in the rank order of fracture probability and the correlation coefficients between surrogate and Singapore Indian versions were close to unity. Thus, an individual at the 90th percentile of risk in Singapore would still be at the 90th percentile of risk using the surrogate FRAX tool. The lower absolute values of probability would, however, become important in the setting of intervention thresholds and in health economic analysis to inform practice guidelines. For example, the use of thresholds derived for Singapore within Pakistan guidelines would have an important impact on the proportion of the population eligible for treatment.

An obvious limitation of this study is the assumption that the fracture rates in Pakistan are similar to Indians living in Singapore. This assumption cannot be tested, and differences between the two populations might impact on this assumption. A high proportion of Indians living in Singapore are from South India (Tamil Nadu) who differ from Pakistanis in many respects that might affect hip fracture risk. In addition to ethnic-specific differences [19], up to twofold differences in hip fracture incidence have been reported using common methodology with the higher rates in urban communities including Croatia [20], Switzerland [21], Norway [22], Argentina [23], and Turkey [24]. Nonetheless, it is of interest that the incidence of hip fracture of Indians in South Africa is very similar to that for Indians living in Singapore [25], which suggests the assumption may not be without credence.

A further limitation, though one shared with the majority of current FRAX models, is that the model was constructed using incidence data on hip fracture only, rather than all major osteoporotic fractures. The latter are calculated from the hip fracture incidence on the basis that the age- and sex-specific relationship between these fractures and hip fractures is similar to that reported in Malmo, Sweden [12]. Importantly, this commonality of pattern has been observed in other studies where data has allowed its assessment [13–15, 26–28].

In summary, a surrogate FRAX model has been created for Pakistan. The model can provide the opportunity to determine fracture probability among the population of Pakistan and help guide decisions about treatment. The latter will require the development of assessment and intervention thresholds. Several approaches have been undertaken to this across practice guidelines worldwide [29]. One such approach, used in more than 50 countries worldwide, bases the intervention threshold on the fracture probability equivalent to a woman with a prior fracture, and is therefore age-dependent [11, 29–35]. If applied to Pakistan, then intervention would be recommended with a probability of a major fracture that varied between 2.1 and 17 % depending on age. The impact of such thresholds or alternative thresholds will require further study.

## Declarations

All procedures performed in studies involving human participants were conducted in accordance with the ethical standards of the institutional and national research committee and with the 1964 Helsinki Declaration and its later amendments or compatible ethical standards.
